# Identification of target genes for RNAi-mediated control of the Twospotted Spider Mite

**DOI:** 10.1038/s41598-018-32742-2

**Published:** 2018-10-02

**Authors:** June-Sun Yoon, Dipak K. Sahoo, Indu B Maiti, Subba R. Palli

**Affiliations:** 1 Department of Entomology, University of Kentucky, Lexington, Kentucky 40546 USA; 2 KTRDC, College of Agriculture, Food and Environment, University of Kentucky, Lexington, KY 40546 USA; 3 Present Address: Department of Agronomy, Iowa State University, Ames, IA 50011 USA

## Abstract

RNA interference (RNAi) is being developed for the management of pests that destroy crops. The twospotted Spider Mite (TSSM), *Tetranychus urticae* is a worldwide pest due to its unique physiological and behavioral characteristics including extraordinary ability to detoxify a wide range of pesticides and feed on many host plants. In this study, we conducted experiments to identify target genes that could be used for the development of RNAi-based methods to control TSSM. Leaf disc feeding assays revealed that knockdown in the expression genes coding for proteins involved in the biosynthesis and action of juvenile hormone (JH) and action of ecdysteroids [Methoprene-tolerant (Met), retinoid X receptor β, farnesoic acid O-methyltransferase, and CREB-binding protein] caused 35–56% mortality. Transgenic tobacco plants expressing hairpin dsRNA targeting *Met* gene were generated and tested. About 48% mortality was observed in TSSM raised on transgenic tobacco plants expressing dsMet. These studies not only broaden our knowledge on understanding hormone action in TSSM but also identified target genes that could be used in RNAi-mediated control of TSSM.

## Introduction

The twospotted spider mite (TSSM), *Tetranychus urticae*, is among the most common cosmopolitan agricultural and garden polyphagous pests^1^. Once the population becomes dense, conventional pest management approaches, such as biological and chemical control often fail to provide relief^2^. These mites have a remarkable ability to quickly develop resistance to toxicants, giving them a greater opportunity to survive on a wide range of host plants and exposure to pesticides^3^. Also, physiological and behavioral characteristics such as a short life cycle, haploid-diploid sex determination, high fecundity, web-spinning behavior, and residing on the underside of leaves have made TSSM control much more complicated^1^.

In arthropods, crosstalk between ecdysteroids and juvenile hormones regulates growth, differentiation, and reproduction by binding to functional heterodimers of proteins that form receptors for these hormones^4^. In insects, 20-hydroxyecdysone binds to a heterodimer of ecdysone receptor (EcR) and ultraspiracle (USP, a homolog of the vertebrate retinoid-X receptor, RXR)^4,5^. In crustaceans, a heterodimer of EcR and RXR functions as an ecdysteroid receptor^5,6^. Methoprene-tolerant (Met) is known for its anti-metamorphic function and has been established as a JH receptor^4^. Besides, the steroid receptor co-activator, SRC is also required for JH signal transduction^7^. CREB-binding protein (CBP) is known to regulate multiple signaling pathways^8^ and interacts with SRC^9^.

In 1976, farnesol, a juvenile hormone (JH) precursor, was detected in whole body homogenates of the deutonymph of TSSM using thin layer chromatography, ultraviolet light analysis, and gas chromatography (GC)-MS^10^. Recently, genes involved in ecdysteroid and JH biosynthesis and action were identified in TSSM^11^. Due to the lack of CYP306A1 and CYP18A1 genes, which encode the biosynthetic enzymes, C25 hydroxylase, and a C26 hydroxylase/oxidase respectively, it has been suggested that the TSSM may use the ecdysteroid, 25-deoxy-20-hydroxyecdysone (ponasterone A, Pon A), as the major molting hormone. This hypothesis was confirmed by biochemical analysis of TSSM extracts using HPLC, enzyme immunoassay and liquid chromatography/mass spectrometry (LC-MS)^11^. Similarly, the absence of CYP15A in the TSSM genome led to a suggestion that methyl farnesoate (MF) could serve as the functional JH^5^. Farnesoic acid O-methyltransferase (FaMet) is the key enzyme involved in catalyzing the final step in the MF biosynthetic pathway in crustaceans^12,13^.

RNA interference (RNAi), a post-transcriptional gene silencing mechanism that helped in studies aimed at elucidating the function of genes. In TSSM two RNAi methods have been demonstrated thus far to deliver the double-stranded RNA (dsRNA): an injection into adult females and eggs, and a leaf-disc feeding assay^14,15^. Injecting *Distal-less* dsRNA into TSSM female adults showed that *Distal-less* phenotype is maternally inherited^14^. The feeding dsRNA method was shown to be a viable method inducing mortality in TSSM after feeding leaf discs treated with dsRNA targeting four known lethal genes^15^, demonstrating the possibility of leaf disc-mediated delivery of dsRNA. Recently. Suzuki and his colleagues compared five different methods to deliver dsRNA to *TSSM*: leaves floating on a dsRNA solution, dsRNA-expressing plants, an artificial diet supplemented with dsRNA, or dsRNA-coated leaves, and mite soaking in dsRNA solution^16^.

The goal of the current study is to identify genes involved in hormone biosynthesis and action and explore their use as RNAi targets in the TSSM. The bean-leaf disc assay was performed to investigate the feeding RNAi effect of eight candidate genes. The transgenic tobacco plants expressing one of the genes, Met, were produced and tested. We also tested JH analogs to determine their effect on TSSM. This information helps in understanding TSSM physiology and also lays a foundation for the development of RNAi-based methods to control this pest.

## Results and Discussion

### Identification of target genes for RNAi-based control of TSSM

**Figure 1:**
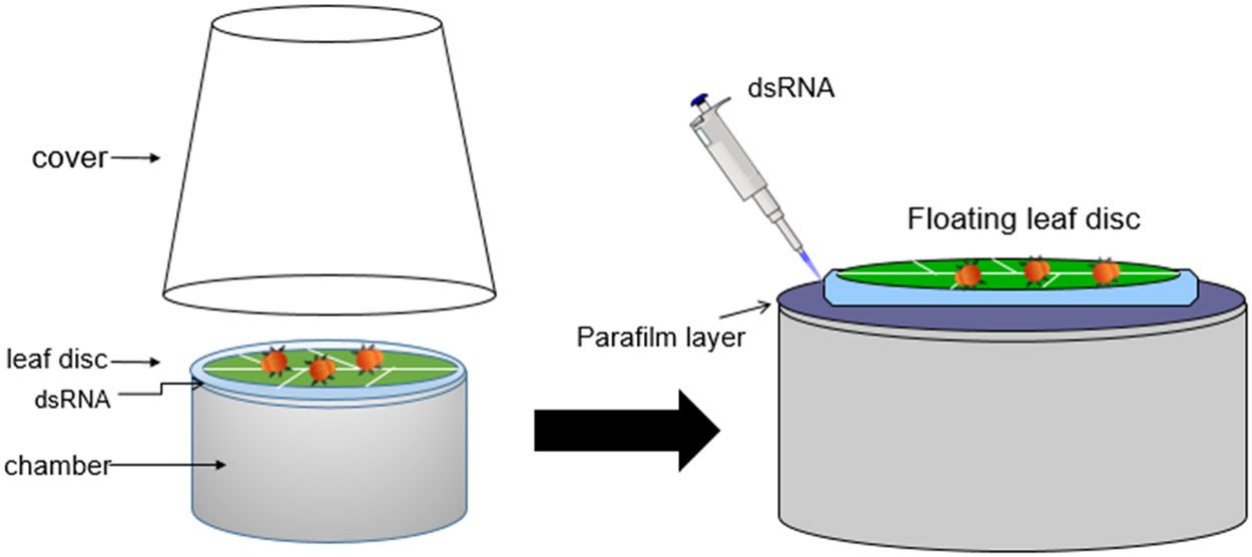
Schematic drawing of the floating bean leaf disc assay. *In vitro* transcribed dsRNAs were delivered to TSSM through bean leaf disc. 200 μl of dsRNA (300 ng/μl concentration) were placed on the leaf on the first day, followed by adding 50 μl of dsRNA on each day up to nine days.

**Figure 2:**
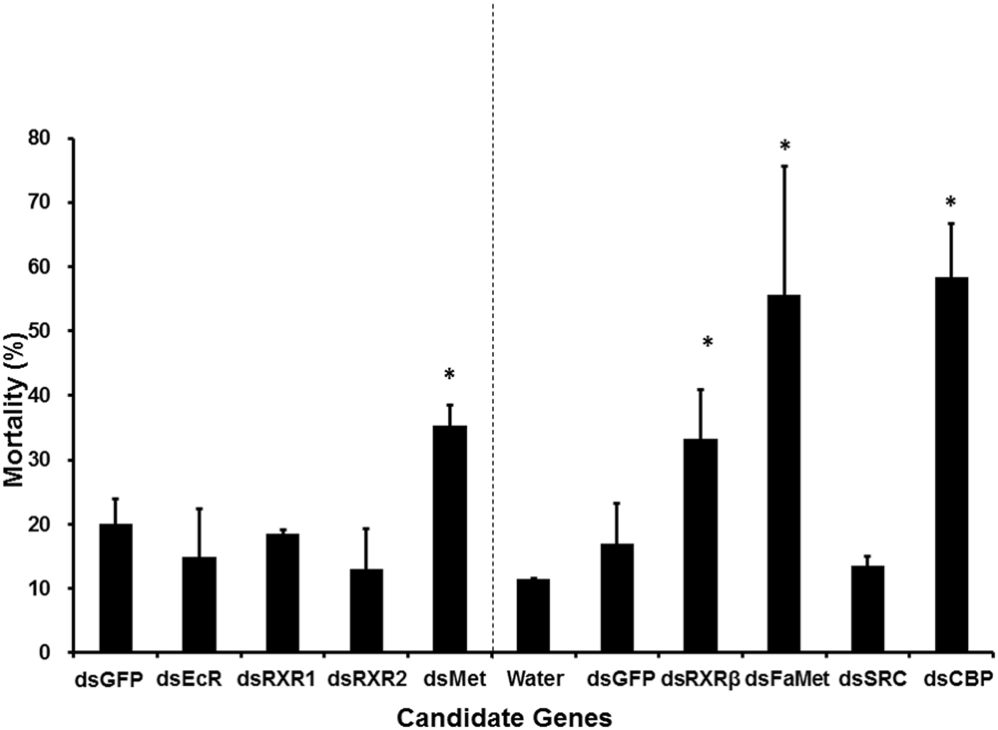
The mortality in TSSM caused by feeding dsRNA targeting genes coding for proteins involved in JH and Ecdysone biosynthesis and action. The dsRNA targeting eight candidate genes coding for proteins involved in JH biosynthesis and action and ecdysteroid action were fed to TSSM. The mortality of TSSM was recorded on the 9^th^ day after application of dsRNA. dsGFP and water were used as controls.

**Figure 3:**
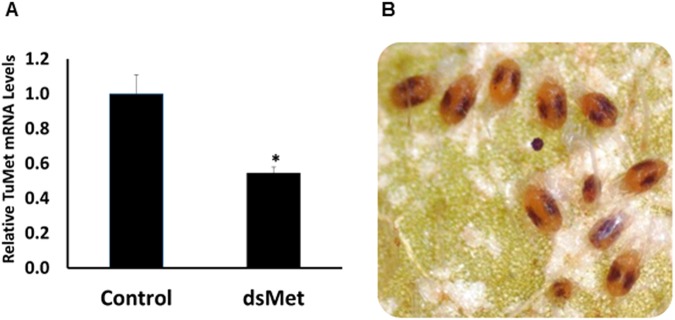
dsMet caused knockdown of Met gene expression and mortality of TSSM. (**A**) dsGFP and dsMet were fed to TSSM for seven days. The TSSM were collected from the bean leaf discs and RNA isolated and used in qRT-PCR to determine the relative Met mRNA levels. The error bars show Mean ± SEM (n = 3). (**B**) The leaf disc assay was performed to check the effect of feeding dsMet on TSSM. The picture was taken on day 9; the majority of TSSM died during the last molt stage after feeding on Met dsRNA.

From the *T. urticae* genome website (http://bioinformatics.psb.ugent.be/orcae/overview/Tetur), we retrieved sequences of eight genes, including TuMet (tetur18g03530), TuSRC (tetur41g00280), and FaMet (tuter13g03250), TuEcR (tetur01g15140), TuRXR1 (tetur31g01930), TuRXR2 (tetur01g09240), TuRXR β (tetur01g09220), and TuCBP (tuter07g03920) coding for proteins involved in biosynthesis and action of JH and action of ecdysteroids (Table 1). Gene-specific primers based on these sequences and DNA isolated from TSSM were used to amplify the 300–500 bp fragment of each gene (Table 2). The PCR products were then used to synthesize dsRNAs, and the dsRNAs were screened in bean leaf disc assay. The bean leaf disc assay developed by Kwon *et al*.^15^ was modified and used to screen candidate genes (Fig. 1). Nuclease-free water and dsRNA targeting a fragment of the gene coding for green fluorescent protein (GFP) dsRNA were used as negative controls to check the undesirable effects of treatment and dsRNA on TSSM. Approximately, 10% and 20–22% mortality was observed in control TSSM treated with nuclease-free water and GFP dsRNA, respectively. Among the dsRNAs tested, dsTuMet, dsTuCBP, dsTuRXR β, and dsTuFaMet caused significantly higher mortality compared to the mortality in the control treatment (Fig. 2). Most of the mortality caused by knockdown of TuMet, TuCBP, TuRXR β, and FaMet occurred during the last molting stage (Fig. 2). Knockdown efficiency was determined in TuMet fed deutonymphs, prior to the last molt stage. This stage was selected because most of the TSSM died during the last molting stage (Fig. 3A,B). Feeding dsMet caused about 50% reduction in Met mRNA levels (Fig. 3A). These data suggest that Met, CBP, RXR β, and FaMet are required for successful completion of development and molting in TSSM and these genes could serve as target sites for development of RNAi-based methods to control this pest.

**Table 1:** List of genes tested.

Target name	Gene ID	Functional annotation
TuMet	tetur18g03530	Methoprene-tolerant homolog
TuEcR	tetur01g15140	Ecdysone Receptor
TuRXR1	tetur31g01930	Retinoid X Receptor 1
TuRXR2	tetur01g09240	Retinoid X Receptor 2
TuRXR β	tetur01g09220	Retinoid X Receptor β
TuSRC	tetur41g00280	Nuclear Receptor Coactivator
TuCBP	tuter07g03920	CREB-binding protein
TuFaMet	tuter13g03250	FA/JHA O-methyltransferase

**Table 2:** Sequences of the primers used in this study.

Primer name	Sequences (5′~3′)	Amplicon length (bp)
GFP_dsRNA_F	CGATGCCACCTACGGCAA	248
GFP_dsRNA_R	TGAAGTTCGAGGGCGACA	
TuEcR_dsRNA_F	AGCTCCAAGACAGCAAGAAG	486
TuEcR_dsRNA_R	GTCTCTGAGGGAGACTCATGTA	
TuMet_dsRNA_F	AAGCATCCACCTCGGACATCTCTT	306
TuMet_dsRNA_R	ATTGCGACTCTGGTGTCAGGGAAT	
TuRXR1_dsRNA_F	TGTCGGGAAGAACGAGATTG	340
TuRXR1_dsRNA_R	CGGGTAACTCGGTGAAATGA	
TuRXR2_dsRNA_F	GAGGAGCGACAACGGAATAA	373
TuRXR2_dsRNA_R	CGGCTTGATGTGCTGAATTAC	
TuRXR β _dsRNA_F	TCCGTTTACCGATGCAAGAA	381
TuRXR β _dsRNA_R	GTGGAACGACTCAAGGGTTAT	
TuSRC_dsRNA_F	CGTGACATGCCGAAGAAGATA	355
TuSRC_dsRNA_R	TACCAAGGGCAGACATAGGA	
TuCBP_dsRNA_F	ACCCAGTCACCCAATGTATC	334
TuCBP_dsRNA_R	AAGATGGTGGTGGAGTGTATC	
TuFaMet_dsRNA_F	GGATTGATGCTGAAGGAGGT	329
TuFaMet_dsRNA_R	ATGAGATCCTTGATGGAAGGTG	
rp49_qRT_F	CTTCAAGCGGCATCAGAGC	105
rp49_qRT_R	CGCATCTGACCCTTGAACTTC	
TuMet_qRT_F	GGTGCGCTCCGATGAAATCAATGT	89
TuMet_qRT_R	AGCCTAAGCTAGCGAACGCAGAAT	
TuMet_Trans_F	AGCAAGCTT ATGGCCACTGAGGAAACAATGG	
TuMet_Trans_R	TCGACTCGAGTTATTGTTTGAGATCTAGTTCGGGT	

T7 promoter sequence TAATACGACTCACTATAGGG was added at the 5′ end of each dsRNA primer.

Sequencing of the TSSM genome and further studies provided important insights into biosynthesis and action of two major hormones, ecdysteroids and juvenile hormones. PonA and MF have been proposed as the major ecdysteroid and JH, respectively. However, not much is known about the action of these hormones especially about the receptors that transduce hormone signals. The methoprene-tolerant has been identified as a JH receptor in insects. It is possible that spider mites may use Met as an MF receptor since chemical structures of JH and MF are nearly identical, except for the absence of an epoxide group^17^. Several recent studies suggested that crustaceans which mainly synthesize the sesquiterpenoid, methyl farnesoate use Met as a receptor for MF^18,19^. Taken together our data and the previous reports suggest that these mites may use Met as an MF receptor. However, determination on whether or not Met is an MF receptor requires further studies. Interestingly, Silencing of TuFaMet, an enzyme involved in MF biosynthesis, also induced mortality of mites confirming previous findings on the role of MF as a major JH in these mites. In insects, homologs of steroid receptor co-activator (SRC) play essential roles in both JH and 20E action^7^. Silencing of SRC homolog in TSSM did not cause significant mortality suggesting that SRC homolog we tested may not be an essential gene in TSSM development. However, further studies are required to determine if there are other homologs of SRC or some other co-activators involved in hormone action in TSSM. Cyclic AMP response binding protein interacts with multiple transcription factors and other proteins that regulate many developmental processes in insects and other animals. Interestingly, knockdown of the CBP gene caused significant mortality in TSSM suggesting that some of the functions of CBP recently shown in insects may have been conserved in TSSM^20,21^. RXR homolog in insects, ultraspiracle, is an essential partner for EcR in transduction of 20-hydroxyecdysone signals in insects. Knockdown of TSSM RXR β also caused significant mortality (Fig. 2). Knockdown of RXR β but not RXR1 and RXR2 caused significant mortality in TSSM suggesting that RXR β is essential for TSSM development. Differences in the effect of RXR β, RXR1 and RXR2 knockdown on TSSM development is intriguing. In insects, USP and RXR isoforms are produced by alternative splicing. The three RXR β and the other two RXRs seem to be coded by different genes in TSSM. Also, RXR1 and RXR2 are typical nuclear receptors containing canonical DNA-binding and ligand-binding domains. Whereas RXR β protein contains a ligand binding domain but not zinc finger-containing DNA binding domain. Structure of RXR1 and RXR2 proteins suggest that they may have redundant function hence, silencing of each of these genes did not show a significant effect on development. Further work on the structure of RXR genes and isoform-specific effects will help to explain the action of RXRs in TSSM. Whether RXR β is required for PonA action or some other function in TSSM requires further studies as well. Lack of mortality after EcR knockdown is also intriguing and requires further investigation. Taken together, the data included here identified Met, CBP, RXR β, and FaMet as potential targets for the development of RNAi-based methods for controlling TSSM.

### Production and testing of transgenic tobacco plants

**Figure 4:**
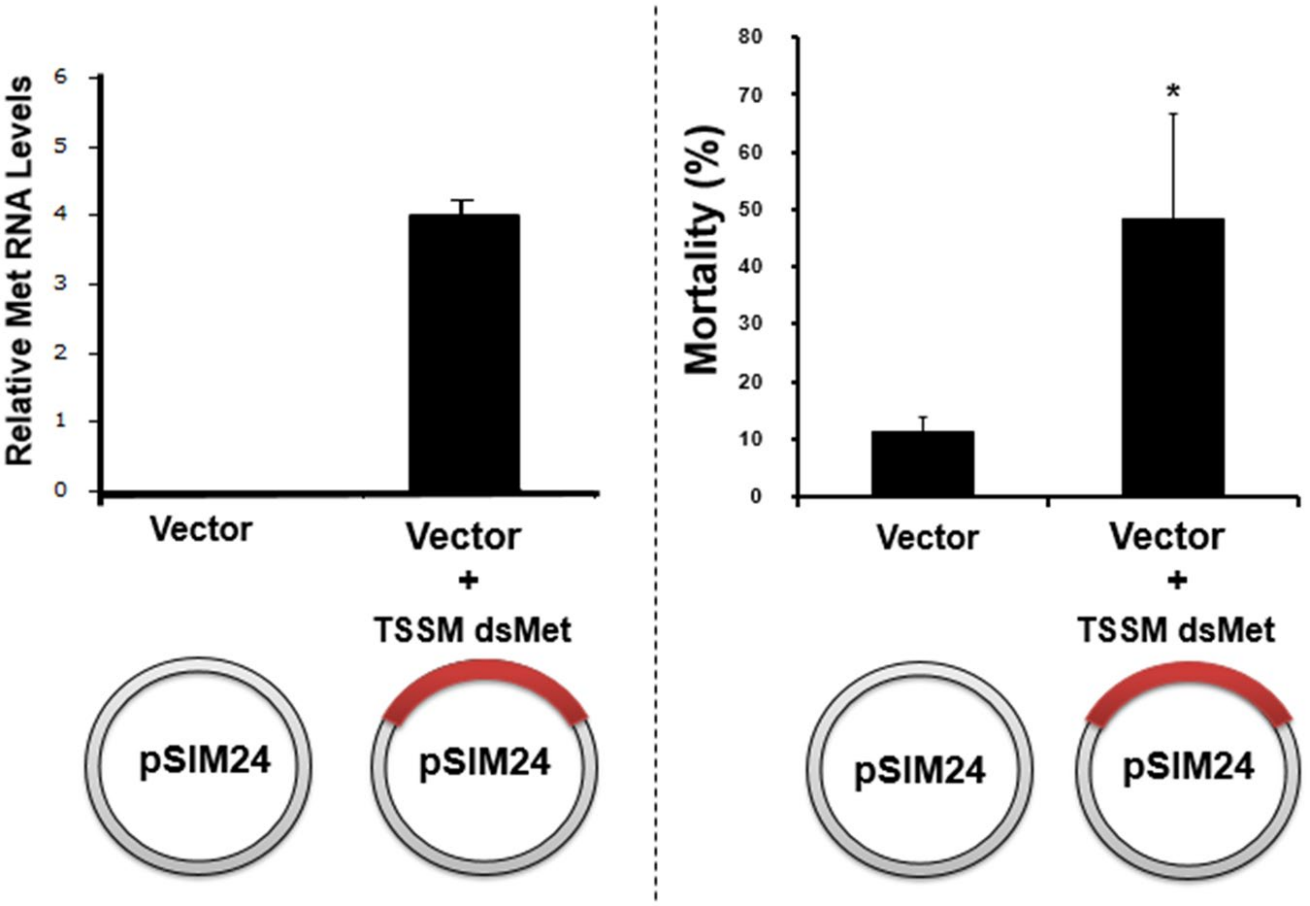
Verification of TuMet expression in transgenic tobacco plants. (**A**) Total RNA isolated from Met transgenic plants and control plants expressing the vector only were used in qRT-PCR to determine TuMet mRNA levels. The error bars show Mean ± SEM (n = 3). (**B**) Tobacco leaves cut into to 4 × 4 cm squares were placed above the wet cotton in a Petri dish. Twenty to thirty TSSM larvae were placed on control and dsMet expressing leaf discs. The mortality was checked on seventh day after initiation of feeding assay. The error bars show Mean ± SEM (n = 3).

Among the four genes whose silencing resulted in significant mortality in the bean leaf disc assay, TuMet was chosen for testing in transgenic plants. A TuMet dsRNA expressing transgenic tobacco plants and the control transgenic plants expressing an empty vector were produced. RNA isolated from transgenic plants and analyzed by qRT-PCR showed the expression of TuMet RNA in transgenic plants but not in control plants (Fig. 4A). These plants were evaluated in TSSM bioassays. About 48% mortality was detected in the TSSM growing on TuMet transgenic plants compared to 10% mortality detected in the TSSM on control plants expressing only vector (Fig. 4B). These data confirm results obtained in leaf disc bioassay and demonstrate the feasibility of delivering dsRNA to TSSM through expression in plants. Poor RNAi efficiency was observed in several feeding assays methods tested in TSSM so far. The methodology/delivery of dsRNA, physiology of TSSM with the highly effective excretory system, and frequent molting during development may have contributed to a decrease in the effectiveness of RNAi in TSSM. Delivering dsRNA to target organisms is one of the challenges associated with developing RNAi-based pest control methods. Some of the approaches that are being developed are expression and delivery through transgenic plants, spraying, soil application or trunk injection of dsRNA synthesized *in vitro* or in microorganisms^22–24^. Several previous studies demonstrated successful delivery of dsRNAs to target insects after their expression in plants^2,20,22,24–27^. When economical and acceptable to the public, this may be the desirable method of delivery of dsRNA to TSSM. Other methods including spray or soil application of dsRNA synthesized *in vitro* or in microorganisms should also be explored for TSSM control.

### Juvenile hormone analogs for TSSM control

Since knockdown of genes involved in JH biosynthesis and action caused mortality in TSSM, we evaluated JH analogs (JHA) for their potential to control TSSM. Insect growth regulators, especially JHAs, have been widely used to control mosquitoes, scales, and mealybugs^28,29^. Here, we tested kinoprene, pyriproxyfen, and hydroprene to see whether they cause mortality in TSSM. Treatment with kinoprene caused significant mortality (88.5%) and blocked development. In contrast, treatment with different concentrations of pyriproxyfen and hydroprene was not effective in blocking development or causing mortality of TSSM (Table 3). In the case of kinoprene treatment, mites mostly died during early stages and did not reach the first molt. Insect growth regulators are widely used for controlling pests all over the world. Very few studies have been performed to determine the effect of JHA on the TSSM. Only one study showed the effectiveness of JHA on TSSM, CGA 29′170 and ZR-777 (kinoprene)^30^. Our results confirm these previous studies where 0.15% ZR-777 caused above 60% mortality on day two and 80% mortality on day four after treatment^30^. The mortality observed in our experiment was around 60~80% on day two after treatment which confirms the previous report. Previous studies identified methoprene as the most effective JHA for *Aedes aegypti*^31^ and hydroprene as the most effective one for *Tribolium castaneum*^26^*;* significant differences in the effectiveness of three JHAs against TSSM is interesting. Taken together these data suggest that JHA work differently among arthropods. Whether or not the differences in JHA activity is due to the differences in Met protein sequence coded by genomes of these arthropods remains to be investigated. The data included in the paper not only broaden our knowledge of hormone action in TSSM but also identified target genes for use in RNAi-based control of TSSM.

**Table 3:** Effect of JHA on the survival and development of *TSSM*.

Juvenoids	Conc.	Solvent	% Reaching maturity^a^	% Mortality
Pyriproxyfen	3 ppm	Water	60.1 ± 14.9	—
	30 ppm	Water	51.9 ± 12.3	—
	60 ppm	Water	62.2 ± 3.5	—
	Control	Water	60.1 ± 8.3	—
Kinoprene	1 %	Acetone	—	88.5 ± 11.5*
	Control	Acetone	—	3.3 ± 1.7
	1 %	Cyclohexane	—	64.3 ± 9.0*
	Control	Cyclohexane	—	12.6 ± 4.9
Hydroprene	1 %	Cyclohexane	—	12.6 ± 3.0
	Control	Cyclohexane	—	12.6 ± 4.9

-No data provided.

*An asterisk denotes a significant difference from the control (student t-test, P < 0.01).

^a^Includes both males and females.

## Materials and Methods

### Twospotted spider mites

Laboratory strain of the twospotted spider mites obtained from John Snyder’s lab at the University of Kentucky was reared on red kidney bean (*Phaseolus vulgaris*)^27^ plants. The TSSM were reared in a fume hood maintained at 23 °C and 55 ± 5% relative humidity.

### Target genes from TSSM genome database

The nucleotide sequences of putative hormone receptors and other genes (Met, SRC, EcR, RXR1, RXR2, RXRβ, FaMet, and CBP) were obtained from the Orca website (http://bioinformatics.psb.ugent.be/orcae/overview/Tetur).

### Total RNA extraction, PCR, dsRNA synthesis and Quantitative real-time PCR (qRT-PCR)

Total RNA was isolated from deutonymphs using the TRI reagent (Molecular Research Center Inc., Cincinnati, OH). DNase I (Ambion Inc., Austin, TX) treated RNA was converted cDNA using M-MLV reverse transcriptase (Clontech Laboratories). The cDNA and the primers designed based on target gene sequences (Table 2) were used to amplify fragments of target genes. The PCR products were purified using the QIAquick PCR purification kit (Qiagen Inc, Valencia, CA) and used as templates to synthesize dsRNA using the Ambion MEGAscript transcription kit (Ambion, Austin, TX). Double-stranded RNA was purified by phenol/chloroform extraction followed by ethanol precipitation. The quality of dsRNAs was checked by running them on agarose gels. The concentration of dsRNAs was measured using NanoDrop1000 spectrophotometer (Thermo Fisher Scientific Inc., Waltham, MA).

The qRT-PCR was performed in Applied Biosystems StepOnePlus™ Real-Time PCR System (Life Technologies™, Carlsbad, CA) using FastStart SYBR Green Master (Roche Diagnostics, Indianapolis, IN). Relative mRNA levels were determined in triplicate biological samples using ribosomal protein 49 (RP49) mRNA levels for normalization.

### Bean leaf disc assay

The feeding chamber and dsRNA-permeated leaf disc were used as described in a previous study^15^ with some modifications to the shape of the chamber and quantity of dsRNA used. The 200 μl of dsRNA (300 ng/μl concentration) was placed underneath the leaf on the first day, followed by adding 50 μl of dsRNA on each day up to nine days. Since the amount dsRNA nearly reached a peak level within the leaf disc by after 24 hr after application^15^, mites were placed on the leaf disc at 24 hr after the application. Twenty-to-thirty larvae were placed on the dsRNA-permeated leaf disc. These chambers were maintained at 23 ± 1 °C, 55 ± 5% relative humidity (RH) at 16:8 (L:D) photoperiod. The green fluorescent protein (GFP) dsRNA was used as a control. When the mites reached adulthood (eight to nine days later), the remaining mites on each leaf disc were counted. The bean leaf disc assay was repeated three times. To determine knockdown efficiency, qRT-PCR was performed using the gene-specific primers (Table 2). A pool of multiple mites were used for each biological replicate and three biological replicates were included in each treatment.

### Transgenic plant production and assay

The transgenic tobacco plants expressing dsRNA targeting TuMet were produced following methods described previously^32^. Tobacco strain (Nicotiana tabacum L. cv. Samsun NN) was used in these experiments. Tobacco plant transformation was done as described previously by Sahoo *et al*.^32^. Tobacco leaf, cut into to 4 × 4 cm squares, were placed above the wet cotton in the petri dish. Twenty to thirty larvae were placed on the tobacco leaf, and mortality was checked on the seventh day after initiation of feeding. Each experiment was repeated three times.

### Statistical analysis

Experimental data were analyzed by Student’s t-test to compare the difference between the control group and treatment group.

## Data Availability

The materials used in the experiments and data reported are available upon request.
